# Silver Cluster‐Porphyrin‐Assembled Materials as Advanced Bioprotective Materials for Combating Superbacteria

**DOI:** 10.1002/advs.202103721

**Published:** 2021-11-10

**Authors:** Man Cao, Shan Wang, Jia‐Hua Hu, Bing‐Huai Lu, Qian‐You Wang, Shuang‐Quan Zang

**Affiliations:** ^1^ Henan Key Laboratory of Crystalline Molecular Functional Materials Henan International Joint Laboratory of Tumor Theranostical Cluster Materials Green Catalysis Center and College of Chemistry Zhengzhou University Zhengzhou 450001 China; ^2^ Laboratory of Clinical Microbiology and Infectious Diseases Department of Pulmonary and Critical Care Medicine National Clinical Research Center for Respiratory Diseases China‐Japan Friendship Hospital Beijing 100029 China; ^3^ China Guangdong Key Laboratory for Emerging Infectious Diseases National Clinical Research Center for Infectious Diseases Shenzhen Third People's Hospital Southern University of Science and Technology Shenzhen 518055 China

**Keywords:** antibiotic‐resistant bacteria, atomically precise silver cluster, charge transfer, photocatalyst, reactive oxygen species (ROS), silver cluster‐porphyrin assembled materials

## Abstract

Superbugs are bacteria that have grown resistant to most antibiotics, seriously threating the health of people. Silver (Ag) nanoparticles are known to exert a wide‐spectrum antimicrobial property, yet remains challenging against superbugs. Here, Ag clusters are assembled using porphyrin‐based linkers and a novel framework structure (Ag_9_‐AgTPyP) is produced, in which nine‐nuclearity Ag_9_ clusters are uniformly separated by Ag‐centered porphyrin units (AgTPyP) in two dimensions, demonstrating open permeant porosity. Ag_9_‐AgTPyP eliminates over 99.99999% and 99.999% methicillin‐resistant *Staphylococcus aureus* (*MRSA*) and *Pseudomonas aeruginosa* (*P. aeruginosa*) within 2 h upon visible‐light irradiation, which are superior to a majority of bacteria inactivation photocatalysts. The novel‐established long‐term charge‐transfer states from AgTPyP to adjacent Ag_9_ cluster that has preferential affinity to O_2_ greatly promote reactive oxygen species (ROS) production efficiency; and its unique framework accelerates the ROS transportation. Personal protective equipment (masks and protective suits) incorporating Ag_9_‐AgTPyP film also shows excellent performances against superbugs. This superbugs‐killing efficiency is unprecedented among silver complexes and porphyrin derivatives. Utilizing efficient photogenerated electrons and holes between metal cluster and linkers can open up new interests of research in photocatalytic areas.

## Graphical abstract

Silver cluster‐porphyrin assembled materials (Ag_9_‐AgTPyP) are demonstrated as advanced bioprotective materials for combating superbacteria, in which the photogenerated charge efficiently transfers from AgTPyP to adjacent Ag_9_ cluster that has preferential affinity to O_2_ and greatly promoting reactive oxygen species production.

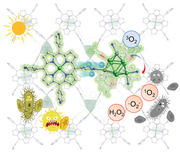

## 1 Introduction

More recently, multidrug‐resistant pathogenic bacteria, also called “superbacteria” or “superbugs,” featuring strong infectiousness and high mortality, have become one of the most serious threats to global safety.^[^ ^1^ ^]^ Some superbacteria that release a large amount of toxins could be utilized as bioweapons via the aerosol route of exposure.^[^ ^2^ ^]^ If these bacteria are delivered successfully in a military context, inevitable soldiers’ casualties and subsequent healthcare delivery system chaos would result. Although personal protective equipment, such as face masks and bioprotective suits could intercept pathogenic bacteria physically, the risk of acquisition of a superbacteria‐related infectious disease for soldiers and healthcare workers who take care of the infectious patients are still high due to the sustained activity of captured superpathogens.^[^ ^3^ ^]^ Silver nanoparticles (AgNPs) as the broad‐spectrum antibacterial materials have been widely used in daily life.^[^ ^4^ ^]^ However, the limited activity toward superbacteria^[^ ^5^ ^]^ and intrinsic toxicity^[^ ^6^ ^]^ is a knotty problem to be settled for advanced bioprotective equipment. Therefore, developing novel bioprotective materials to efficiently prevent the transmission of superbacteria‐related infectious diseases is of utmost urgency, yet remains a formidable challenge.

Combating bacteria via photocatalysis strategies has attracted increasing attention because of its high biocidal efficiency.^[^ ^3^, ^7^ ^]^ In particular, photoinduced antimicrobial materials hold great promise for the constructing bioprotective equipment.^[^ ^3^, ^7^ ^]^ Porphyrins and metalloporphyrins with strong visible light‐harvesting and oxygen transport abilities are promising photosensitizers for antibacterial photocatalytic therapy via reactive oxygen species (ROS) production, such as peroxide, superoxide, hydroxyl radicals, and singlet oxygen.^[^ ^8^ ^]^ Nevertheless, they easily aggregate and could result in self‐quenching issues.^[^ ^9^ ^]^ One approach to overcome this issue is to embed porphyrins into metal organic framework scaffolds, which provide good accessibility for ROS generation and transportation due to the well‐developed porous structure and isolated sites.^[^ ^8^, ^10^ ^]^

Herein, we use a porphyrin‐based linker to assemble Ag clusters, which have well‐defined structures and much smaller sizes than Ag nanoparticles, producing a novel framework structures ([Ag_9_(*^t^* BuC≡C)_6_(CF_3_COO)_3_(AgTPyP)]*_n_*, Ag_9_‐AgTPyP), in which nine‐nuclearity Ag_9_ clusters are uniformly separated by Ag‐centered porphyrin units (AgTPyP) in two dimensions. Particularly, Ag_9_‐AgTPyP integrated light harvesters, silver sites, and high surface areas all in one catalyst with an orderly manner, simultaneously optimizing the photocatalytic kinetics for efficient ROS production and leading to high superbacterial inactivation efficiency. As a result, Ag_9_‐AgTPyP showed outstanding photocatalytic inactivation efficiency against superbacteria methicillin‐resistant *Staphylococcus aureus* (*MRSA*, >99.99999%) and *Pseudomonas aeruginosa* (*P. aeruginosa*, >99.999%) under visible light within 120 min at a catalyst dose of 50 mg L^−1^. Theoretical calculations and ultrafast transient absorption (TA) spectroscopy suggested that the porphyrinic unit in Ag_9_‐AgTPyP behaves as an antenna to harvest visible light, leading to formation of the excited state, which then transfers electrons to the catalytic sites around the Ag_9_ cluster, enabling the activation of O_2_ to various ROS, including singlet oxygen (^1^O_2_), superoxide anion (·O_2_^−^), and hydrogen peroxide (H_2_O_2_). More importantly, we fabricated Ag_9_‐AgTPyP film as the bioprotective layer and incorporated into the face masks or bioprotective suits, which displayed intriguing photoactive antibacterial performance in both aerosol and liquid forms, demonstrating their great potential as bioprotective materials against superbacteria.

## 2 Results and Discussion

**Figure 1:**
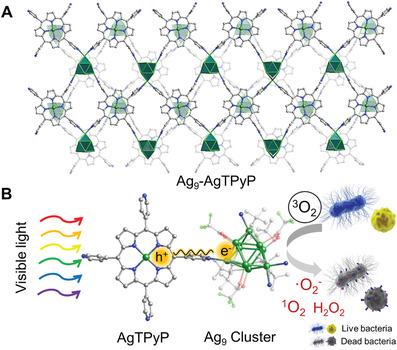
Ag_9_‐AgTPyP photocatalytic inactivation of bacteria. A) Perspective view of the Ag_9_‐AgTPyP crystal structure. B) Schematic representation of Ag_9_‐AgTPyP photocatalytic inactivation of antibiotic‐resistant bacteria.

Ag_9_‐AgTPyP was synthesized by a one‐pot reaction of TPyP, CF_3_COOAg, and AgC≡C*^t^* Bu in a mixed solution of dimethylformamide (DMF) and CHCl_3_ via a conventional slow solvent evaporation method. Single‐crystal X‐ray diffraction analysis revealed that Ag_9_‐AgTPyP crystallizes in the *C2/c* space group (Table S1, Supporting Information), in which the 4‐connected Ag_9_ node is linked with µ_4_‐TPyP ligands to form a 2D framework that adopts an AB stacking mode (**Figure** **1**A,B and Figure S1, Supporting Information). Of note, one Ag atom was spontaneously incorporated into the free‐base TPyP ligand to form the metalloporphyrin AgTPyP. Such in situ metallization at the porphyrin core may provide more accessible metal centers to achieve a synergistic enhancement of antimicrobial activity. The phase purity of the bulk Ag_9_‐AgTPyP product was confirmed by a comparison between the simulated and experimental powder X‐ray diffraction (PXRD) patterns (Figure S2, Supporting Information). The core of the Ag_9_ cluster is a tower‐like structure, capped by six *^t^* BuC≡C^−^ anionic ligands with two kinds of mixed *σ*‐type and *π*‐type bonding modes, namely, µ_3_‐*ηπ*_1_, *ηπ*_1_, *ησ*_1_ *2 and µ_3_‐*ηπ*_2_, *ηπ*_1_, *ησ*_1_ *4, and three CF_3_COO^−^ ligands with µ_2_‐*η*_1_, *η*_1_ bonding types (Figures S3 and S4, Supporting Information). The Ag_9_ core is further consolidated by numerous inner close Ag(I)···Ag(I) contacts, with distances of 2.8823(16)–3.0901(12) Å (Table S2, Supporting Information). These distances are shorter than the sum of the van der Waals radii of two silver ions (3.44 Å), suggesting the presence of argentophilic interactions.^[^ ^11^ ^]^ Moreover, the chemical composition of the structure was further confirmed by infrared spectroscopy (Figures S5, Supporting Information). Thermogravimetric analysis (TGA) curve indicated that Ag_9_‐AgTPyP was thermally stable at 117 °C (Figures S6, Supporting Information).

Compared to the isolated silver clusters, the highly assembled materials provide enhanced stability by resisting the attack of various guest species.^[^ ^12^ ^]^ The crystallinity of Ag_9_‐AgTPyP was well maintained after immersing the samples in water for 24 h or exposing them to air for 5 months (Figure S2, Supporting Information). Ag_9_‐AgTPyP is hydrophobic, with a water contact angle of 115.6°, which is attributed to the exposed terminal ‐CF_3_ and ‐*^t^* Bu groups in the Ag_9_ cluster (Figure S7, Supporting Information). Such hydrophobic surfaces could efficiently block the adhesion of bacteria and prevent microbial colonization on the surface.^[^ ^13^ ^]^ The permeant porosity of Ag_9_‐AgTPyP was confirmed by nitrogen sorption measurements at 77 K, the Brunauer–Emmett–Teller specific surface area was determined to be 190 m^2^ g^−1^, which provides good accessibility for O_2_ encapsulation and ROS production (Figures S8, Supporting Information). In addition, Ag oxidation states in Ag_9_‐AgTPyP were studied by X‐ray photoelectron spectroscopy (XPS). Ag 3d_5/2_ and 3d_3/2_ peaks were observed at binding energies of 368.2 and 374.2 eV, respectively, indicating the presence of both Ag(II) and Ag(I) oxidation states in Ag_9_‐AgTPyP (Figure S9, Supporting Information). The +2 oxidation state of Ag originates from the porphyrin macrocycle and is obtained during the in situ insertion process, while the +1 oxidation state mainly exists in the Ag_9_ cluster.^[^ ^14^ ^]^

**Figure 2:**
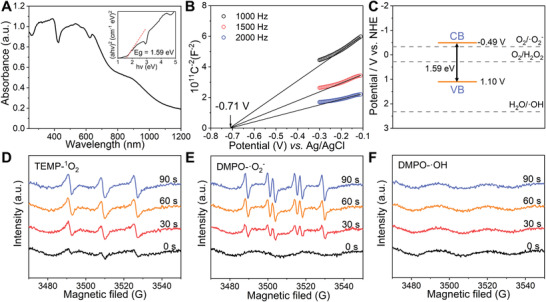
Band‐structure characterization and ROS generation of Ag_9_‐AgTPyP. A) UV‐Vis spectra and determined optical energy gap of Ag_9_‐AgTPyP. B) Mott–Schottky plots of Ag_9_‐AgTPyP in a 0.2 m Na_2_SO_4_ aqueous solution. C) The band positions of Ag_9_‐AgTPyP with respect to the ROS formation potential. EPR spectra of Ag_9_‐AgTPyP for testing of D) ^1^O_2_, E) ·O_2_^−^, and F) ·OH in an air atmosphere under visible light irradiation.

The UV‐Vis diffuse reflectance spectra of Ag_9_‐AgTPyP showed strong absorption over a wide range from 240 to 800 nm due to the light‐harvesting porphyrinic macrocycle (**Figure** **2**A). According to the Tauc plot, the band gap energy was estimated to be 1.59 eV (Figure 2A). Furthermore, the conduction band (CB) position of Ag_9_‐AgTPyP was estimated by measuring the flat‐band potential via Mott–Schottky measurements, which were performed at frequencies of 1000, 1500, and 2000 Hz (Figure 2B). The positive slopes of the C^−2^ values versus potential plot indicate that Ag_9_‐AgTPyP is an n‐type semiconductor and that most of the carriers are electrons. The CB was determined by fitting to be −0.71 V versus Ag/AgCl (i.e., −0.49 V vs normal hydrogen electrode (NHE)), and the corresponding valence band (VB) was calculated to be 1.10 V versus NHE. Additionally, the VB potential was determined by using valence band X‐ray photoelectron spectroscopy (VB‐XPS) (Figure S10, Supporting Information), and the result was consistent with the Mott–Schottky result. Accordingly, we illustrated the band structure of Ag_9_‐AgTPyP in Figure 2C. Since the CB was more negative than the oxygen reduction potential of O_2_/·O_2_^−^ (−0.33 V vs NHE, pH 7) and O_2_/H_2_O_2_ (0.28 V vs NHE, pH 7),^[^ ^7^, ^15^ ^]^ Ag_9_‐AgTPyP meets the thermodynamic requirements for the generation of ·O_2_^−^ and H_2_O_2_ (Figure 2C). In addition, we analyzed the O_2_ sorption ability of Ag_9_‐AgTPyP, which showed an O_2_ uptake of 51.69 cm^3^ g^−1^ at 77 K (Figure S8, Supporting Information). Considering its wide‐range light‐harvesting ability, proper band alignment, and high O_2_ uptake, Ag_9_‐AgTPyP is an ideal candidate for photocatalytic ROS generation.

In our previous study, we demonstrated that the synergistic effect of silver clusters and porphyrin units contributed to the transformation of ground‐state oxygen (^3^O_2_) to ^1^O_2_.^[^ ^12b^ ^]^ Herein, the production of ^1^O_2_ in Ag_9_‐AgTPyP was confirmed by using electron paramagnetic resonance (EPR) with 2,2,6,6‐tetramethyl‐4‐piperidinol (TEMP) as a spin probe, and a typical 1:1:1 triplet peak for 4‐hydroxy‐2,2,6,6‐tetramethylpiperidine 1‐oxyl (TEMPOL) was observed upon light illumination (Figure 2D). The ·O_2_‐ generation was examined by utilizing trapping reagent 5,5‐dimethyl‐1‐pyrroline N‐oxide (DMPO), which displayed the specific quartet signals of DMPO‐·O_2_‐ with an intensity ratio of 1:1:1:1 (Figure 2E). Moreover, the amount of ^1^O_2_ and ·O_2_^−^ production gradually increased as the irradiation time was increased from 0 to 30, 60, and 90 s. The steady‐state concentration of ^1^O_2_ produced by Ag_9_‐AgTPyP was measured by testing the decay of furfuryl alcohol (FFA) and was determined to be (2.91 × 10^−7^) × 10^−6^ m (Figure S11, Supporting Information). The concentration of steady‐state ·O_2_^−^ was determined to be (1.80 × 10^−4^) × 10^−6^ m by the nitroblue tetrazolium (NBT) reduction method (Figure S12, Supporting Information). Additionally, we investigated the production of ·OH during the photocatalysis process, but no signals were detected (Figure 2F), which explained the more positive potential of H_2_O/·OH (2.32 V vs NHE, pH 7)^[^ ^7^, ^15^ ^]^ than of the VB of Ag_9_‐AgTPyP. Moreover, the generation of H_2_O_2_ was monitored by using a fluorescent method with *N*‐acetyl‐3,7‐dihydroxyphenoxazine (Amplex Red) as an indicator, which can be oxidized to luminescent resorufin in the presence of horseradish peroxidase (HRP). After 120 min of irradiation, the H_2_O_2_ concentration in this system was determined to be 3.40 × 10^−6^ m (Figure S13, Supporting Information). The H_2_O_2_ concentration in the Ag_9_‐AgTPyP system was much higher than that of ^1^O_2_ and ·O_2_^−^, and previous literature has reported that H_2_O_2_ has the strongest bacterial inactivating effect.^[^ ^7a–c^ ^]^ Overall, the produced ROS species could oxidize the lipid bilayer of bacteria and react with proteins and other cell components, causing bacterial death.

**Figure 3:**
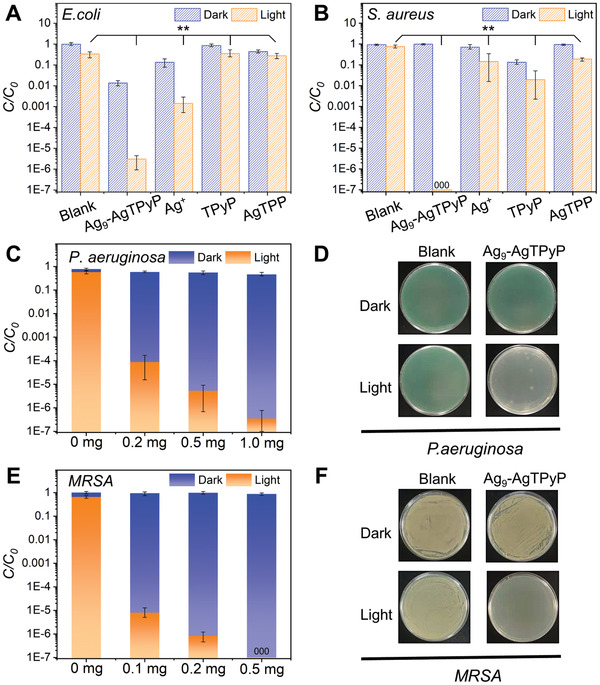
Antibacterial properties of Ag_9_‐AgTPyP. Comparison of the photocatalytic antibacterial performance of Ag_9_‐AgTPyP, Ag^+^, TPyP, and AgTPP under both light and dark conditions on A) *E. coli* and B) *S. aureus* (**p < 0.01). C) Inactivation efficiency of different amounts of Ag_9_‐AgTPyP toward the antibiotic‐resistant bacteria *P. aeruginosa*. D) Photos of plate count agars spread with *P. aeruginosa* before and after photocatalytic disinfection using Ag_9_‐AgTPyP (0.5 mg). E) Inactivation efficiency of different amounts of Ag_9_‐AgTPyP toward the antibiotic‐resistant bacteria *MRSA*. F) Photos of plate count agars spread with *MRSA* before and after photocatalytic disinfection using Ag_9_‐AgTPyP (0.5 mg). In the graph of inactivation performance, all bars represent group means. Error bars indicate maximum positive deviation and maximum negative deviation of the mean. *p*‐Values were calculated using one‐way analysis of variance (ANOVA) (*n* = 3). The data marked by three zeros (000) on the bar indicate that no live bacteria were detected.

The photocatalytic antibacterial performance of Ag_9_‐AgTPyP was initially tested against two representative bacterial strains, gram‐positive *S. aureus* and gram‐negative *E. coli*. The photocatalytic bacterial inactivation experiments were conducted in 10 mL 0.9% w/v saline at an initial bacteria density of 10^8^ colony‐forming units (CFU) mL^−1^ using 50 mg L^−1^ photocatalyst and irradiation by a white light‐emitting diode (LED) lamp with a UV filter (>420 nm). As shown in Figure S14 in the Supporting Information, both *E. coli* and *S. aureus* were significantly reduced within 120 min, suggesting that Ag_9_‐AgTPyP is an excellent broad‐spectrum antimicrobial material. Impressively, the inhibition efficiency reached over 99.999% (equivalent to −log_10_(*C/C_0_*) = 5) for *E. coli* at 120 min and 99.99999% (equivalent to −log_10_(*C/C_0_*) = 7) for *S. aureus* at 90 min, which are much higher than the results presented in many previous reports on materials such as porphyrin‐based coordination polymers^[^ ^8^ ^]^ and typical semiconductor‐based materials^[^ ^7^, ^16^ ^]^ (Table S3, Supporting Information). Furthermore, the time‐dependent bacterial change curves indicated that the antibacterial efficiency of Ag_9_‐AgTPyP toward *S. aureus* was much faster and higher than that of toward *E. coli*. The difference in inactivation performance on *E. coli* and *S. aureus* may be ascribed to the distinct chemical composition and structure of the bacterial membrane.^[^ ^17^ ^]^ Furthermore, scanning electron microscopy (SEM) images were used to visualize the morphologies of the treated bacteria. Obviously, the membranes of *E. coli* collapsed and ruptured compared with those of the control groups, while *S. aureus* retained its membrane integrity (Figure S15, Supporting Information). In addition, we noticed that Ag_9_‐AgTPyP showed an inactivation efficiency of ≈1 log toward *E. coli* even in the dark (**Figure** **3**A), which was probably caused by the leaching out Ag ions from Ag_9_‐AgTPyP. The inductively coupled plasma optical emission spectrometry (ICP‐OES) results showed a trace amount of Ag^+^ (0.24 ppm, 1.12%) leaching after photocatalytic bacterial inactivation. Accessible Ag ions may be coordinated by sulfhydryl groups in proteins to deactivate cellular enzymes and DNA, leading to bacterial death.^[^ ^17^ ^]^ In a control study, we utilized CF_3_COOAg with the same amount of Ag^+^, which showed lower antibacterial efficiency against *E. coli* (−log_10_(*C/C_0_*) = 2, ≈99.00%) and *S. aureus* (−log_10_(*C/C_0_*) < 1, ≈85.63%) at 120 min than did Ag_9_‐AgTPyP (Figure 3A,B), illustrating that the ROS generated by Ag_9_‐AgTPyP mainly affected the bacteria. Moreover, the TPyP ligand alone and Ag‐metallized TPP (denoted as AgTPP) were tested under identical test conditions for comparison, and both showed inactivation efficiencies of <1 log for *E. coli* (<91.75%) and *S. aureus* (<99.68%) (Figure 3A,B and Figure S16, Supporting Information). These results confirm that the distinctive scaffold frameworks of Ag_9_‐AgTPyP are crucial to the antibacterial activity. PXRD pattern of Ag_9_‐AgTPyP is well retained after 2 h antibacterial process, suggested that Ag_9_‐AgTPyP was stable during the photocatalytic process (Figure S17, Supporting Information).

Encouraged by these results, we explored the bacterial inactivation ability of Ag_9_‐AgTPyP on superbacteria. *P. aeruginosa* and *MRSA* have been identified by the World Health Organization (WHO) as priority pathogens that are fatal to humans and require an extended therapy time of 20 days.^[^ ^13^ ^]^ The photosensitizing antibacterial performance is shown in Figure 3C. More than 99.9999% (−log_10_(*C/C_0_*) = 6) of *P. aeruginosa* were killed after 120 min of irradiation with 1 mg of Ag_9_‐AgTPyP, and the elimination efficiency toward *MRSA* reached 99.99999% (−log_10_(*C/C_0_*) = 7) with 0.5 mg of Ag_9_‐AgTPyP. This impressive antibacterial activity motivated us to decrease the catalyst concentration. With 0.2 mg of Ag_9_‐AgTPyP, an elimination efficiency of ≈4 log for *P. aeruginosa* (>99.99%) and 6 log for *MRSA* (>99.9999%) were observed, and an inactivation efficiency of more than 5 log for *MRSA* (>99.999%) was achieved in 120 min even with 0.1 mg catalyst (Figure 3C–F and Figure S18, Supporting Information). These results strongly demonstrated that Ag_9_‐AgTPyP has an excellent photocatalytic antibacterial effect on superbacteria.

Additionally, SYTO9/propidium iodide (PI) staining was used to perform the live/dead tests. As shown in Figure S19 in the Supporting Information, most bacteria are alive in the control groups that shown negligible fluorescence, indicating few bacteria dead. On the contrary, upon incubating with Ag_9_‐AgTPyP and irradiation, obvious red fluorescence on all kinds of bacteria were observed, further suggesting that Ag_9_‐AgTPyP is a photoactive antibacterial agent. Besides, the toxicity of Ag_9_‐AgTPyP in vitro was investigated. The result indicated that the cell viabilities of HeLA cells remained over 95% at the dose test (80 mg L^−1^), suggesting low toxicity of Ag_9_‐AgTPyP (Figure S20, Supporting Information).

**Figure 4:**
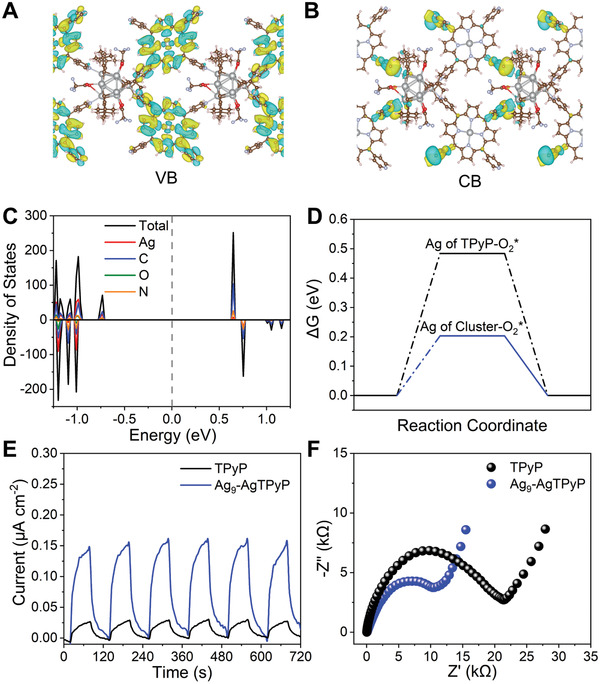
Theoretical calculations and charge transfer efficiency for Ag_9_‐AgTPyP. A) The top of the VB and B) the bottom of the CB of Ag_9_‐AgTPyP calculated by VASP. C) Calculated DOS profiles of Ag_9_‐AgTPyP. D) Free‐energy diagram of O_2_ adsorption on active Ag metal sites in AgTPyP (black line) and the Ag_9_ cluster (blue line). E) Photocurrent responses and F) EIS Nyquist plots for Ag_9_‐AgTPyP and TPyP.

The remarkable superbacterial inactivation efficiency of Ag_9_‐AgTPyP was mainly ascribed to effective ^1^O_2_, ·O_2_^−^, and H_2_O_2_ species generation. It is well known that the efficient separation of photoinduced electron–hole pairs is crucial to ROS generation and photocatalytic performance.^[^ ^7a,b^ ^]^ To disclose the roles of the Ag_9_ cluster subunit and AgTPyP ligand in the photoexcited charge‐carrier separation mechanism, we carried out density functional theory (DFT) calculations in the Vienna Ab initio Software Package (VASP). **Figure** **4**A,B reveals that the electrons occupying the top of the VB in Ag_9_‐AgTPyP are centered on the porphyrin unit. However, the bottom of the CB moved toward the center of the silver clusters, suggesting that charge transfer occurred from the excited porphyrin to the Ag_9_ cluster when Ag_9_‐AgTPyP was excited by light (Figure 4B,C). Moreover, the adsorption energy for the binding of O_2_ to the catalyst was calculated to further understand the electron transfer process (Figure 4D). The results show that the binding of O_2_ on the Ag_9_ cluster is stronger than the binding on Ag ions in the porphyrin, which is favorable for the activation of O_2_ to ROS. In addition, the EPR spectra also provide some information on the reaction pathway.

The charge transfer efficiency of Ag_9_‐AgTPyP was initially examined by light on–off photoelectrochemical and electrochemical impedance spectroscopy (EIS) experiments. As displayed in Figure 4E,F, Ag_9_‐AgTPyP showed a higher photocurrent response and lower charge transfer resistance than TPyP, indicating that the Ag_9_ cluster node in the extended framework could efficiently accelerate electron transfer and prohibit electron–hole recombination. Furthermore, the significantly quenched photoluminescence (PL) intensity of Ag_9_‐AgTPyP relative to TPyP confirms that the separation of electron–hole pairs is improved (Figure S21, Supporting Information). Another piece of evidence comes from time‐resolved PL decay spectroscopy measurements (Figure S21, Supporting Information). Compared to isolated TPyP (*τ* = 9.14 ns), Ag_9_‐AgTPyP showed a much shorter lifetime (*τ* = 7.65 ns).

**Figure 5:**
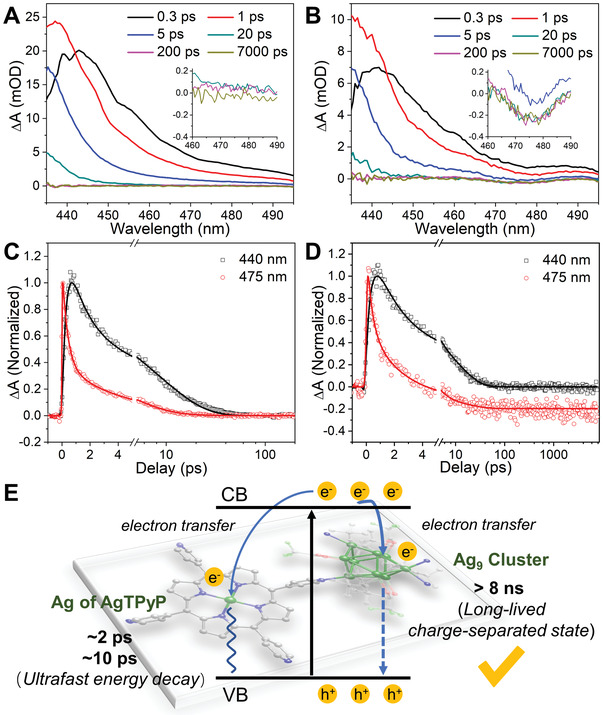
TA measurements. The TA spectra (pump at 520 nm) of A) AgTPP and B) Ag_9_‐AgTPyP taken at different probe delays. Representative TA kinetics of C) AgTPP and D) Ag_9_‐AgTPyP taken at the probing wavelength of 440 nm (black line) and 475 nm (red line). E) Mechanisms underlying the photoexcited dynamics involved in Ag_9_‐AgTPyP.

Further insight into the rapid charge separation in Ag_9_‐AgTPyP was obtained using transient absorption (TA) spectroscopy. In **Figure** **5**A,B, the TA spectra of both AgTPP and Ag_9_‐AgTPyP at 1 ps showed a new increased excited state absorption at 440 nm compared with that at 0.3 ps, and the kinetic signal corresponding to probe wavelength of 440 nm showed a rising signal, indicating that energy transfer from porphyrin to its central silver was occurred (Figure 5C,D). While, the process is ultrafast with only ≈300 fs, and the energy decay occurs rapidly with a short lifetime (≈2 ps, ≈10 ps). Noting that the TA spectra of Ag_9_‐AgTPyP showed a bleaching peak centered at 475 nm in the 460–490 nm range (Figure 5B), which can be ascribed to the fast charge transfer from TPyP to the Ag_9_ cluster. As shown in Figure 5D, the kinetic spectrum at 475 nm indicated that the signal is gradually turning to negative at 4 ps and converges to an asymptote, illustrating that this is a long‐lived state generated after the electron transfer from TPyP to Ag_9_ cluster (with the charge separated state lifetime >8 ns). We speculated that the slow charge recombination process in Ag_9_‐AgTPyP was account for the trapping of electrons in Ag_9_ cluster, and the holes are remained at TPyP (Figure 5E). Besides, the control experiment of TPyP showed unchanged TA spectral signatures at 430–500 nm, suggesting that there was no energy transfer process (Figure S22, Supporting Information). The above spectroscopic analysis demonstrated that the introduction of Ag_9_ cluster could facilitate charge separation by suppressing the detrimental electron–hole combination.

Consequently, the generated ROS concentration of Ag_9_‐AgTPyP was much higher than that of the ligand TPyP, as determined by 3,3′,5,5′‐tetramethylbenzidine (TMB) oxidation experiments (Figure S23, Supporting Information). Ag_9_‐AgTPyP showed strong UV absorbance for the TMB oxidation product as well as a distinct color change from colorless to blue, which suggests a higher oxidation degree of TMB, whereas TPyP exhibited much less activity and a slight color change. Various scavengers, including carotene, mannite, catalase, and superoxide dismutase (SOD), were introduced into the system to clearly identify the active oxygen species of ^1^O_2_, ·OH, H_2_O_2_, and ·O_2_^−^. For TPyP, only carotene suppressed the oxidation of TMB, indicating that ^1^O_2_ as the ROS promotes the reaction. In comparison, the TMB oxidation by Ag_9_‐AgTPyP was inhibited by carotene, SOD and catalase, in accordance with our EPR and fluorescence results. The above results are consistent with the fact that Ag_9_‐AgTPyP exhibits a higher disinfection rate than TPyP.

**Figure 6:**
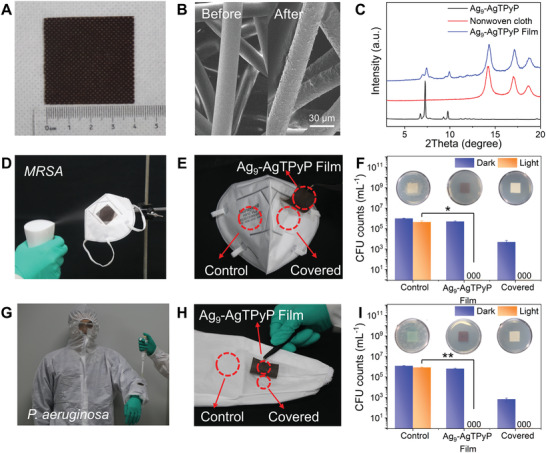
Bioprotection performance of Ag_9_‐AgTPyP film. A) Optical photograph of Ag_9_‐AgTPyP film (4 × 4 cm^2^). B) SEM images of Ag_9_‐AgTPyP film. C) PXRD patterns of Ag_9_‐AgTPyP, nonwoven cloth, and Ag_9_‐AgTPyP film. D) Simulated bacterial aerosols and the interception test by N95 mask. E) Three selected test areas on the mask and F) the relevant CFU count of *MRSA*, its illustration represents each area of mask washed three times incubated in nutrient agar after 1 h of light irradiation (*p < 0.05). G) Photograph showing the protective suit was loaded with Ag_9_‐AgTPyP film. H) Three selected test areas on the protective suit and I) the relevant CFU count of *P. aeruginosa*, its illustration represents each area of mask washed three times incubated in nutrient agar after 1 h of light irradiation (**p < 0.01). All bars represent group means. Error bars indicate maximum positive deviation and maximum negative deviation of the mean. *p*‐Values were calculated using two‐tailed independent student's *t*‐test method (*n* = 3). The data marked by three zeros (000) on the bar indicate that no live bacteria were detected.

Inspired by above results, we fabricated the Ag_9_‐AgTPyP film through a facile hot‐pressing method^[^ ^7^, ^18^ ^]^ as bioprotective layer to defeat superbacteria. Considering that nonwoven fabrics was usually employed as the outermost layer in the personal protective equipment to contact the superbacteria. First, we choose it as substrate to load Ag_9_‐AgTPyP particles (**Figure** **6**A). SEM images and elemental mapping analysis indicated the obtained flexible film coating with uniformly dispersed Ag_9_‐AgTPyP particles, which ranging from 0.5 to 5 µm (Figure 6B and Figure S25, Supporting Information). The loading level was 0.75 mg cm^−2^. Moreover, the PXRD patterns of Ag_9_‐AgTPyP film maintained well with pristine Ag_9_‐AgTPyP (Figure 6C). To examine the applicability of Ag_9_‐AgTPyP film in personal protective equipment, Ag_9_‐AgTPyP film was first integrated into the masks serving as the biocidal layer. As shown in Figure 6D‐F, no living bacteria were observed on Ag_9_‐AgTPyP film area or its covered area in the mask under 1 h of visible light irradiation when the gram‐positive model *MRSA* aerosols was about 10^6^ CFU. On the contrary, most of the bacteria in control area (outer layers of N95 masks) were survived with a negligible antimicrobial efficiency of 54.85%. When the experiment was conducted in dark conditions, the inferior antibacterial activity (48.29%) is not enough to defeat the bacterial infection, further supporting that photocatalytic is a prerequisite to kill superbacteria for Ag_9_‐AgTPyP film. Similarly, Ag_9_‐AgTPyP film also displayed promising bioprotection against gram‐negative model *P. aeruginosa* in liquid form to the protective suits (Figure 6G‐I). Almost no viable bacterial *P. aeruginosa* colonies were observed within 1 h in the covered area upon treatment with Ag_9_‐AgTPyP film under light illumination. While over 10^5^ CFU of bacteria survived in the control group of commercial protective suit surface under the same conditions. Furthermore, the recycling experiment of Ag_9_‐AgTPyP film was conducted. Results showed that no performance decay was observed after three cycles and the PXRD patterns were well maintained, suggesting that the film have great reusability (Figure S26, Supporting Information). Above results strongly supported that Ag_9_‐AgTPyP film showed great potential as a protective layer for combating superbacteria in various scenarios like individual combat equipment in the war, surgical masks, etc.

## 3 Conclusion

In summary, we developed a new silver‐porphyrinic cluster‐assembled material Ag_9_‐AgTPyP, and explored its bioprotection application toward the superbacteria under visible light. A mechanistic investigation indicated that the co‐contribution of silver clusters and porphyrinic units in the framework could efficiently enhance the charge separation ability upon photoexcitation and activate O_2_ to produce ^1^O_2_, ·O_2_^−^, and H_2_O_2_. To be specific, a novel long‐term charge‐transfer state from AgTPyP to adjacent Ag_9_ cluster has been established. We also proved that Ag_9_ cluster has preferential affinity to O_2_ which greatly promoted ROS production efficiency. This research provides a deep understanding of combating multidrug‐resistant bacteria via photocatalysis in silver‐porphyrinic cluster‐assembled materials. Utilizing efficient photogenerated electrons and holes between metal cluster and linkers would open up new interests of research in photocatalytic areas. Considering the tailorable optical and electronic structure of silver clusters and the diversity of organic ligands, we expect that there will be more work on regulating the catalytic ability of silver cluster‐assembled materials.

## 4 Experimental Section

### Synthesis of Ag_9_‐AgTPyP

AgC≡C*^t^* Bu (0.020 g, 0.106 mmol) and CF_3_COOAg (0.022 g, 0.1 mmol) were dissolved in DMF (6 mL), and the solution was stirred for 5 min. Then, 1 mL of trichloromethane solution containing TPyP (0.01 g, 0.016 mmol) was added under stirring, and the solution was subsequently filtered. The filtrate was slowly evaporated in air to give dark‐purple crystals of Ag_9_‐AgTPyP (34.55% yield based on TPyP). Elemental analysis (%) for evacuated Ag_9_‐AgTPyP (C_82_H_78_Ag_10_F_9_N_8_O_6_, M = 2521.21): calcd. C: 39.06, H: 3.09, N: 4.44, O: 3.81; found C: 37.16, H: 2.81, N: 4.52, O: 4.24.

### Synthesis of Ag‐TPP

Ag‐TPP (a complex of 5,10,15,20‐tetraphenylporphyrin chelated with silver ions) samples were synthesized according to a previous report.^[^ ^19^ ^]^

### ROS Measurements

The ^1^O_2_ steady‐state concentration was calculated by testing the decay of FFA using high‐performance liquid chromatography on an Agilent 1100 Infinity instrument with a Supelcosil LC‐18‐DB column (25 cm × 4.6 mm, 5 µm particle size). FFA was dispersed using an isocratic mobile phase (80% acetonitrile and 20% phosphoric acid, 0.1%, pH 3.75) at 1 mL min^−1^ and detected using UV absorbance at 218 nm. The rate constant for the ^1^O_2_ and FFA reaction is 1.2 × 10^8^ M^−1^ s^−1^.^[^ ^20^ ^]^ The ·O_2_^−^ steady‐state concentration was calculated by measuring the decay of NBT using UV‐vis spectroscopy. NBT had an absorption peak at 259 nm. The rate constant for the ·O_2_^−^ and NBT reaction was 5.9 × 10^4^ M^−1^ s^−1^.^[^ ^20^ ^]^ The H_2_O_2_ concentration was measured using an Amplex Red fluorescence probe. The fluorescence of the product was monitored, where the excitation wavelength was 560 nm and the emission wavelength was 582 nm.^[^ ^21^ ^]^ For the EPR test, Ag_9_‐AgTPyP (5 mg) was dispersed in 10 mL of solution (water was used for ^1^O_2_ and ·OH, and methanol was used for ·O_2_^−^) under ultrasonic oscillation for 5 min. Then, 100 µL of the mixed solution was collected, and 100 µL of a spin trapping agent (TEMPO (100 × 10^−3^ m) was used for ^1^O_2_, and DMPO (100 × 10^−3^ m) was used for ·O_2_^−^ and ·OH) was added. EPR signals were recorded on an electron paramagnetic resonance spectrometer under a 300 W xenon lamp (*λ* > 420 nm).

### Photocatalytic Antibacterial Activity Study

Gram‐negative (*E. coli* (ATCC 8739) and *P. aeruginosa* (from clinical isolation)) and gram‐positive (*S. aureus* (ATCC 6538) and *MRSA* (ATCC 43300)) bacteria were used as model bacteria. All vessels and materials were sterilized in an autoclave before the experiments. The bacterial cells were grown in Luria‐Bertani (LB) broth at 37 °C for 18 h to yield a cell count of ≈10^9^ CFU mL^−1^. Then, the bacterial cells were collected by centrifugation (5000 rpm for 10 min) and resuspended in a sterile saline solution (0.9% w/v). The bacterial concentration for the bactericidal study was 10^8^ CFU mL^−1^, which was adjusted by the gradient dilution method using 0.9% w/v saline solution. Typically, 0.5 mg of catalyst was added to a 60 mL photoreactor containing 10 mL of bacterial solution (10^8^ CFU mL^−1^). The bacterial solution and photocatalyst were mixed at room temperature and simultaneously irradiated by white LED light coupled with a 420 nm cut‐off filter for 120 min at a density of 80 mW cm^−2^. As the reaction proceeded, the mixture was carefully pipetted out at scheduled intervals, and the residual bacterial concentrations were determined by the standard plate count method. The plates were incubated at 37 °C for 20 h. The number of colonies was determined through visual inspection. A series of experiments were conducted in the dark under the same conditions used for the dark controls. The light control group was studied in the absence of photocatalyst. No degradation of Ag_9_‐AgTPyP was observed in saline solution over the time range examined in this study. For comparison of the photocatalytic antibacterial performance, the dose of Ag^+^ was equivalent to the leakage amount of Ag_9_‐AgTPyP. Moreover, the doses of TPyP and AgTPP were equivalent to the molar quantities of Ag_9_‐AgTPyP.

### Theoretical Calculations

All the calculations were performed within the DFT framework as implemented in the Vienna Ab initio Software Package (VASP 5.3.5) code, with the Perdew–Burke–Ernzerhof generalized gradient approximation and the projected augmented wave method.^[^ ^22^ ^]^ The cut‐off energy for the plane‐wave basis set was set to 400 eV. The Brillouin zone of the bulk unit cell was sampled by Monkhorst–Pack (MP) grids for Ag_9_‐AgTPyP optimizations.^[^ ^23^ ^]^ The Ag_9_‐AgTPyP was determined by a 1 × 1 × 1 Monkhorst−Pack grid. The convergence criterion for the electronic self‐consistent iteration and force was set to 10^−5^ eV and 0.01 eV Å^−1^, respectively.

### Fabrication of Ag_9_‐AgTPyP Film

12 mg Ag_9_‐AgTPyP was dispersed in 250 µL polyethylene glycol (PEG, *M*_n_ = 400) and 750 µL ethanol. After manually ground, the mixture was loaded on 4 × 4 cm^2^ by heating plate at 100 °C for 5 min. The film was washed by ethanol, and the falling Ag_9_‐AgTPyP powder was collected by centrifugation. Then, the falling powder was again mixed with PEG and ethanol, and coated on the nonwoven cloth through heating plate. The process was repeated until all the powder was loaded onto the film. Finally, Ag_9_‐AgTPyP film was dried in a vacuum oven at 60 °C for 6 h.

### Antimicrobial Assays of Ag_9_‐AgTPyP Film

100 µL of bacterial suspension (*MRSA* or *P. aeruginosa*, 10^7^ CFU mL^−1^) was sprayed or dripped on the surface of control film (outer layer of the N95 mask or protective clothing) and Ag_9_‐AgTPyP film in a size of 2 × 2 cm^2^, and then exposed to white LED light coupled with a 420 nm cut‐off filter or dark conditions for 1 h. After the antimicrobial tests, Ag_9_‐AgTPyP film was fully washed with 0.9 mL of 0.9% w/v saline solution. Then, 100 µL of solution was serially diluted to be plated on nutrient agar culture medium. These plates were incubated at 37 °C for 20 h and the viable cell count was performed to obtain the results for disinfection. Then, filter freshly washed by 10 mL of 0.9% w/v saline solution was cultured in nutrient agar for 20 h at 37 °C for residual analysis of adhered viable cells.

### Statistical Analysis

Data were expressed as mean value from at three independent experiments. Error bars represent the maximum positive deviation and maximum negative deviation. The difference between two groups was analyzed by two‐tailed independent student's *t*‐test method. The differences among multiple groups were analyzed by one‐way analysis of variance (ANOVA). *p* < 0.05 was considered to be statistically significant with noting via * (** represents *p* < 0.01). All statistical analyses were performed using SPSS 23 software.

[CCDC 2054440 contains the supplementary crystallographic data for this paper. These data can be obtained free of charge from The Cambridge Crystallographic Data Centre via www.ccdc.cam.ac.uk/data_request/cif.]

## Conflict of Interest

The authors declare no conflict of interest.

## Supporting information

Supporting Information

## Data Availability

Research data are not shared.
