# Supplementary material for: Silver Cluster‐Porphyrin‐Assembled Materials as Advanced Bioprotective Materials for Combating Superbacteria
Source: Adv Sci (Weinh). 2021 Nov 10;9(2):2103721. doi: 10.1002/advs.202103721 (PMC8805555; doi:10.1002/advs.202103721)
Supplement: Supplementary file 1 — Supporting Information [file ADVS-9-2103721-s001.pdf]

WILEY-VCH

© 2021 Wiley-VCH GmbH

ADVANCED SCIENCE
Open Access

Supporting Information

for *Adv. Sci.,* DOI: 10.1002/advs.202103721

Silver cluster-porphyrin assembled materials as advanced bioprotective materials for combating superbacteria

Man Cao, Shan Wang, Jia-Hua Hu, Bing-Huai Lu, Qian-You Wang and Shuang-Quan Zang**

Supporting Information

Silver cluster-porphyrin assembled materials as advanced bioprotective materials for combating superbacteria

Man Cao[†]*, Shan Wang*[†]*, Jia-Hua Hu, Bing-Huai Lu, Qian-You Wang* and Shuang-Quan Zang**

Supplementary Text

Materials and measurements

All reagents and solvents were commercially available and used as received. Non-woven fabric employed in this work is non-woven fabric with polypropylene (PP). Its density is 0.31 g cm^{-3}. Powder X-ray diffraction (PXRD) patterns of the samples were obtained in the range of 3-50° on a Riguku D/Max-2500PC X-ray diffractometer with Cu Kα radiation (λ = 1.54178 Å) at 298 K. The optical absorption and diffused reflectance spectra were obtained on a Hitachi UH4150 spectrophotometer. Single-crystal X-ray diffraction (SCXRD) was performed on a Rigaku XtaLAB Pro diffractometer using Cu Kα radiation (λ = 1.54184 Å). X-ray photoelectron spectroscopy (XPS) was performed on a Thermo ESCALAB 250XI electron spectrometer using 300 W Al Kα radiation. The C peak at 284.6 eV was used as a reference to correct for charging effects. Scanning electron microscopy (SEM) images were obtained using a Zeiss Sigma 500. The electrochemical performance and photoelectrochemical performance were measured in a three-electrode quartz cell system using a platinum plate as the counter electrode, a Ag/AgCl electrode as the reference electrode, and a Ag_9-AgTPyP-coated platinized carbon electrode as the working electrode. The electrolyte was 0.2 M Na_2SO_4. The photoelectrochemical results were recorded using a CHI660E electrochemical station. Visible light irradiation was achieved by using an 80 mW/cm^2 white LED using Perfect Light PCX-50C (λ > 420 nm). Electron paramagnetic resonance (EPR) signals were recorded with a Bruker A300 spectrometer. ICP-TOFMS signals were obtained using an Optimass 9500. Confocal images were taken with LEICA TCS SP8 STED fluorescence confocal microscopy system.

In Vitro Cytotoxicity Assay

Cell Counting Kit-8 (CCK-8) assays were carried out to estimate the cytotoxicity of Ag_9-AgTPyP. HeLa cells were cultured in RPMI-1640 medium with 10% FBS and 1% penicillin-streptomycin at 37°C, 5% CO_2. Briefly, HeLa cells were cultured in a 96-well plate at a density of 1×10^4 cells per well (100 µL medium/well). After cultivation overnight, the old medium was replaced with 100 µL fresh medium containing different concentrations of Ag_9-AgTPyP (0, 5, 10, 20, 40, 80, 160 mg L^{-1}) for another 12 h incubation. Then, the cells were washed with DPBS completely and incubated in 100 µL of culture medium containing 10% CCK-8 at 37 °C for additional 1 h. The plate was softly shaken for 30 s and then the absorbance at 450 nm was measured on a microplate reader.

Live/Dead Cell Fluorescence Assay

Above differentially processed bacteria within 0.9% (w/v) saline solution were collected by centrifugation with 8000 rmp respectively. The collected bacteria cells were stained in dark by incubating with 1.5 µL mL^{-1} SYRTO9 and 1.5 µL mL^{-1} PI together for 30 min. Then, 10 µL of bacterial solution was dropped onto a glass slide. Confocal images were taken with LEICA TCS SP8 STED fluorescence confocal microscopy system (excitation: 488 and 552 nm).

Transient absorption (TA) measurements

The femtosecond TA data were recorded on a modified pump-probe spectrometer (Helios Fire, Ultrafast Systems LLC) in combination with an ultrafast laser system (Coherent). The 520 nm pump pulse at ~150 μJ/cm^2 was delivered by an optical parametric amplifier (OPA). The white-light continuum (WLC) probe pulses (430-760 nm) were generated by focusing the 800 nm beam onto a sapphire plate. The pump-probe delay was controlled by a optical delay line. The temporal and spectral profiles (chirp-corrected) of the pump-induced differential transmission of the WLC probe light (i.e., absorbance change) were visualized by an optical fiber-coupled multichannel spectrometer (with a CMOS sensor). The 500 nm shortpass fliter was inseted before the detector to remove the pump pulse so that only 430-500 nm probe range were detected.

3,3',5,5'-Tetramethylbenzidine (TMB) Oxidation Measurements

Typically, 3 mg of TMB was dispersed with 1 mL of H_2O and 2 mL of HAc/NaAc buffer solution (1:1, 0.1 M). Ag_9-AgTPyP (2.50 mg, 1 μmol), TPyP (0.62 mg, 1 μmol) and AgTPP (0.72 mg, 1 μmol) was then added into the mixture solution under white LED light (>420 nm) irradiation respectively. The samples were taken at different time intervals using the function of UV-vis absorption plot record on Microplate Reader. In order to verify the specific ROS, various scavengers were added into the TMB solution before the light irradiation: carotene (2 mg), mannite (2 mg), catalase (1 mg), and superoxide dismutase (SOD, 0.5 mL), respectively.

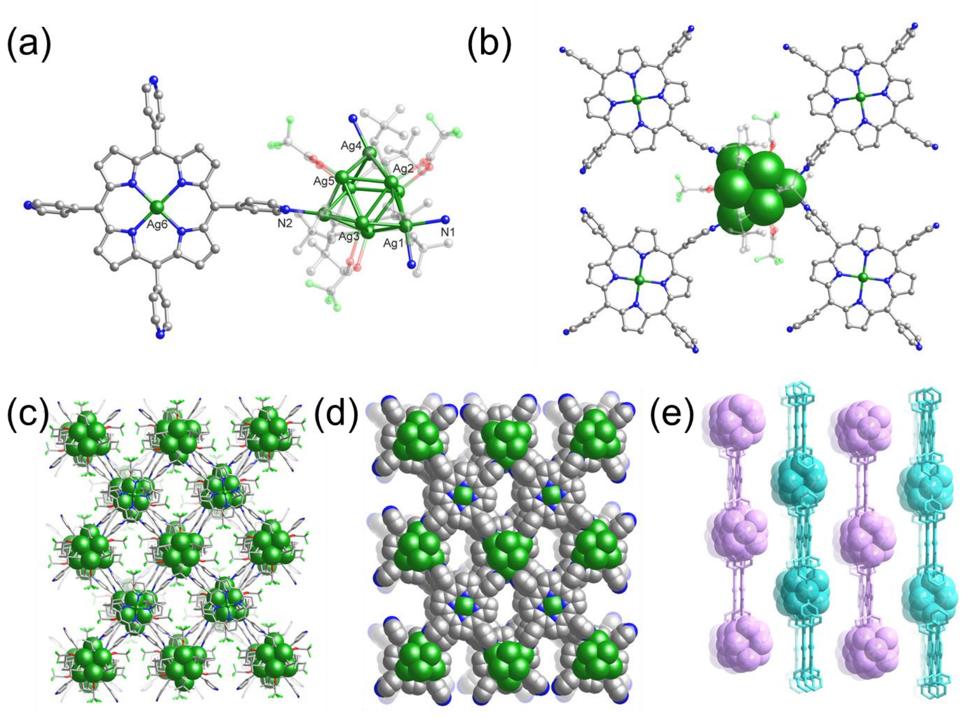

Figure S1. Summary of structural information about Ag_9-AgTPyP: (a) The coordination environments of the Ag(I) centres in Ag_9-AgTPyP. Colour code: Ag, dark green; F, green; O, red; N, blue; C, grey. Symmetry codes: #1 1-X, +Y, 1/2-Z; #2 +X, -1+Y, +Z; #3 1-X, -1+Y, 1/2-Z; #4 3/2-X, 3/2-Y, 1-Z; #5 +X, 1+Y, +Z (b) Different coordination sites of two kinds of nitrogen atoms. (c) A 3D (4, 4) connected framework was formed by the 4-connected AgTPyP linkers and Ag_9 clusters. (d) 3D spacing stacking and (e) twofold AB packed structure.

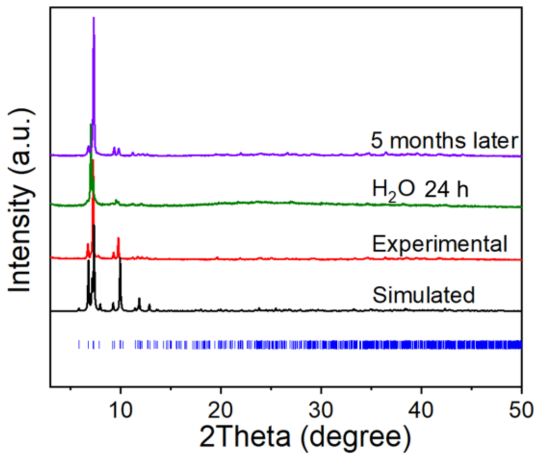

Figure S2. PXRD patterns of Ag_9-AgTPyP under water conditions for 24 h and 5 months later.

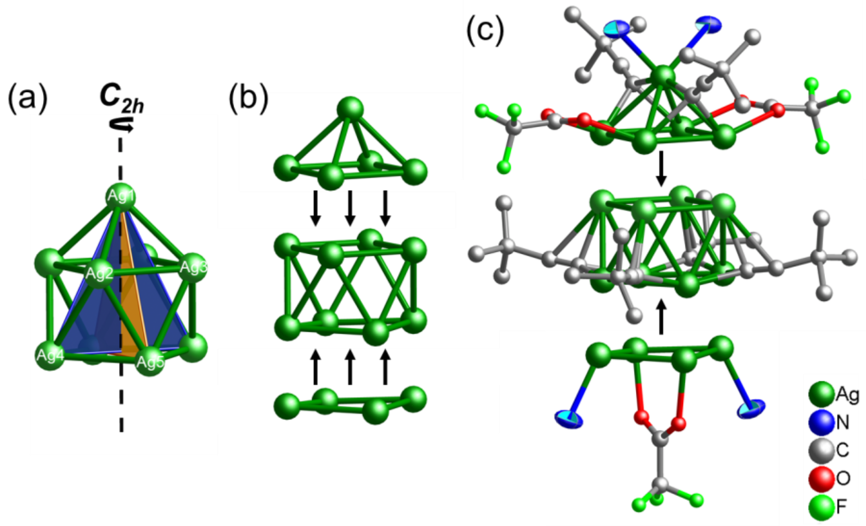

Figure S3. Summary of structural information about the Ag_9 core of Ag_9-AgTPyP: (a) the tower-like Ag_9 core of the cluster with a C_{2h} axis of symmetry. (b) The anatomy of the Ag_9 core. (c) The core protected by the ligand. Colour code: Ag, dark green; F, green; O, red; N, blue; C, grey.

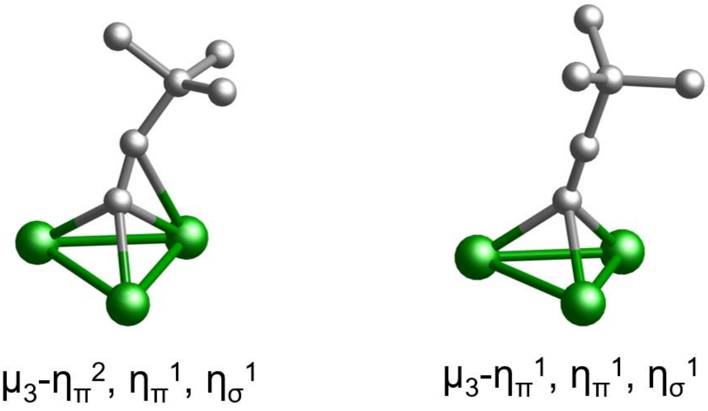

Figure S4. The coordination modes of $^{t}BuC{\equiv}C^{-}$ in Ag_9-AgTPyP.

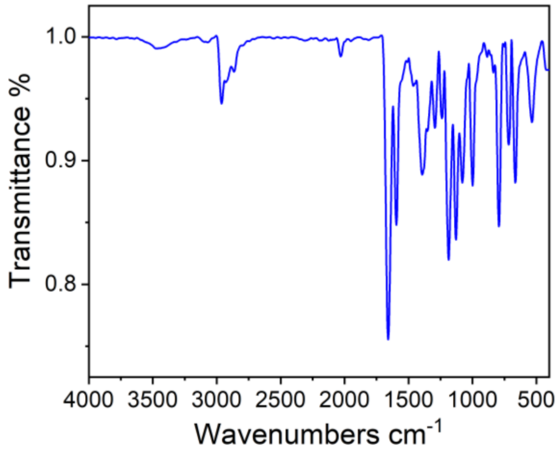

Figure S5. Infrared spectrogram of Ag_9-AgTPyP.

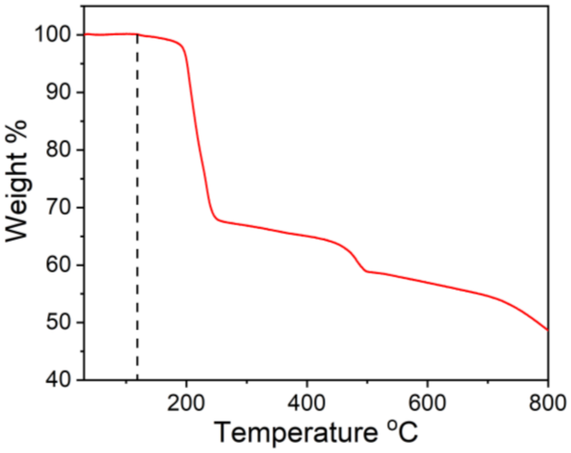

Figure S6. The TG curve of Ag_9-AgTPyP.

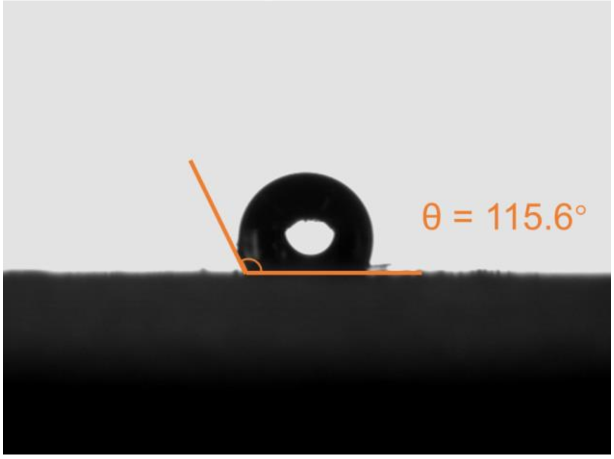

Figure S7. Water contact-angle of Ag_9-AgTPyP.

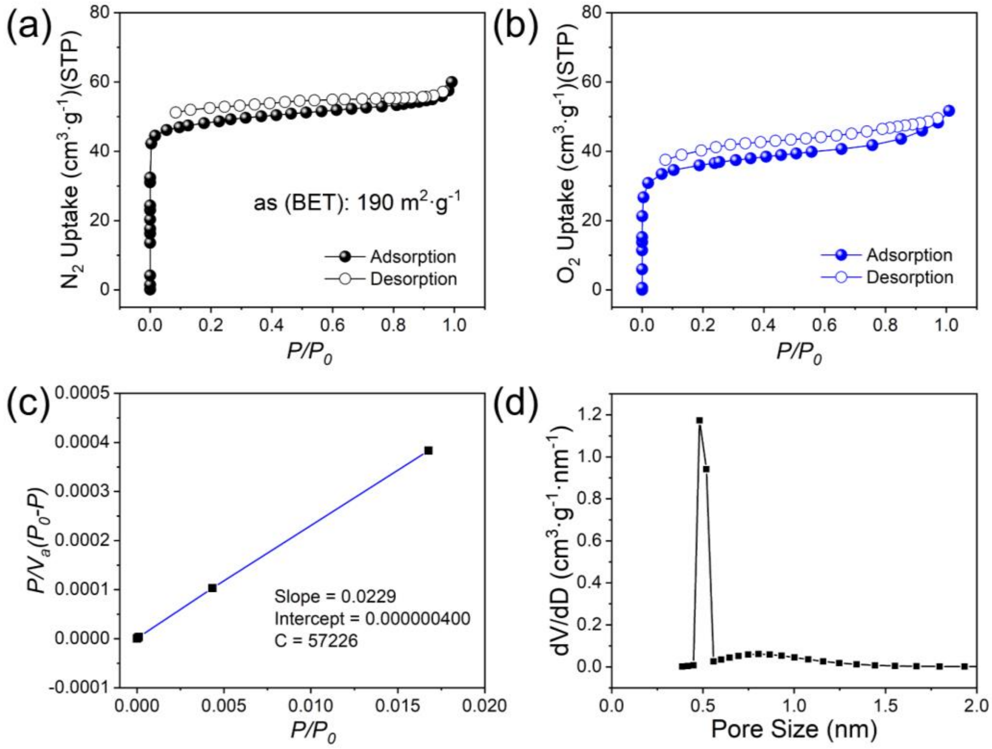

Figure S8. N_2 (a) and O_2 (b) adsorption isotherms of Ag_9-AgTPyP at 77 K. (c) Selected BET Plots of Ag_9-AgTPyP for BET surface area calculation and constant C based on N_2 adsorption isotherm at 77 K. (d) Pore size distribution profile based on nonlocal density function theory (NLDFT) of Ag9-AgTPyP.

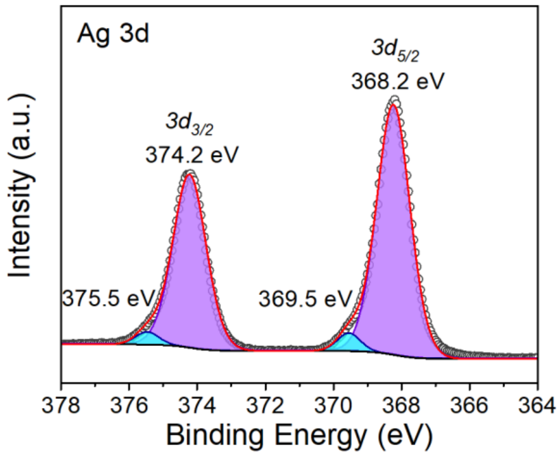

Figure S9. Ag 3d XPS spectra of Ag_9-AgTPyP.

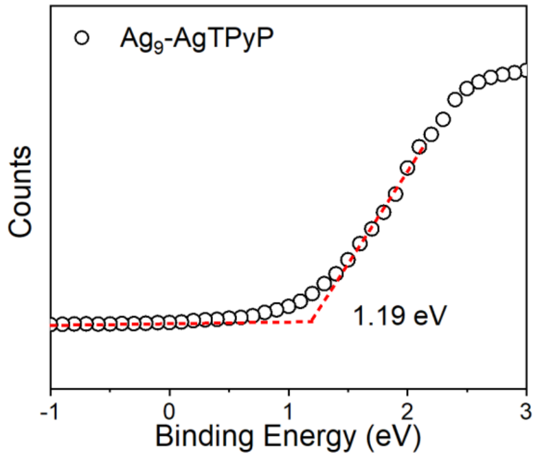

Figure S10. VB-XPS spectra of Ag_9-AgTPyP. The energy scales were aligned by using the Fermi level of the XPS instrument (4.35 eV versus the absolute vacuum value).

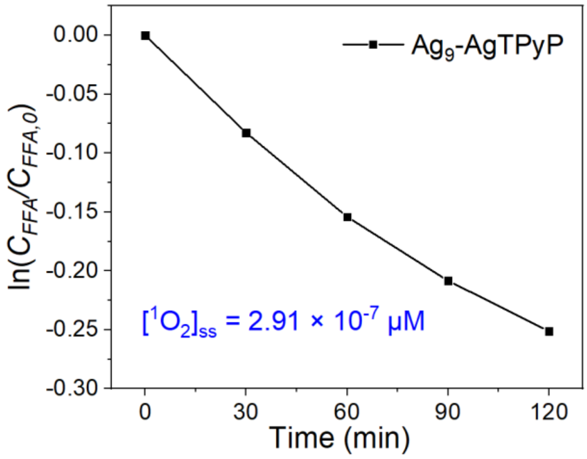

Figure S11. Steady state concentration of $[^1O_2]_{ss}$ calculated from the decay of furfuryl alcohol (FFA) of Ag_9-AgTPyP.

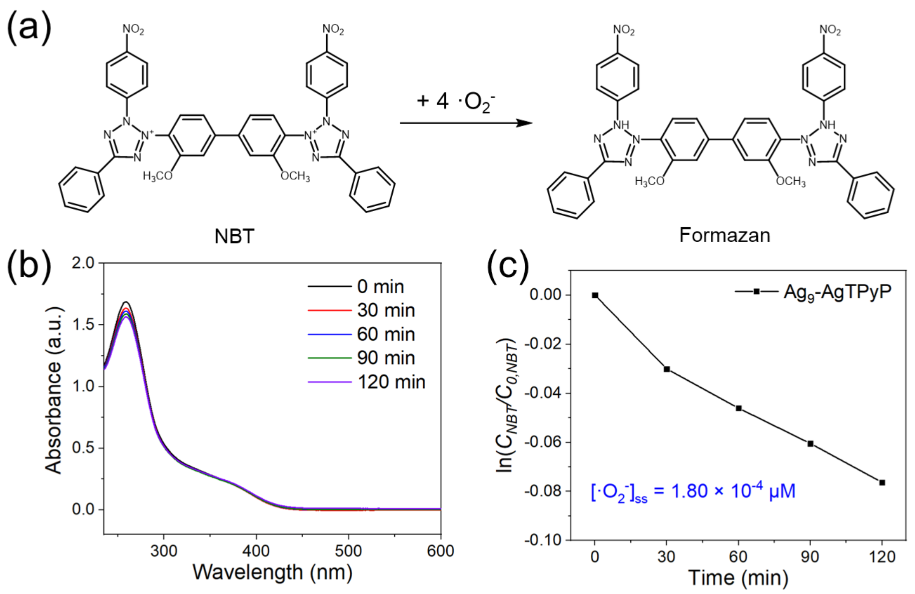

Figure S12. (a) Equations for the decay reaction of nitroblue tetrazole (NBT). (b) Time-dependent absorption spectra of NBT for $\cdot O_2^-$ detection by Ag_9-AgTPyP. (c) Steady-state concentration of $[\cdot O_2^-]_{ss}$ calculated from the decay of NBT.

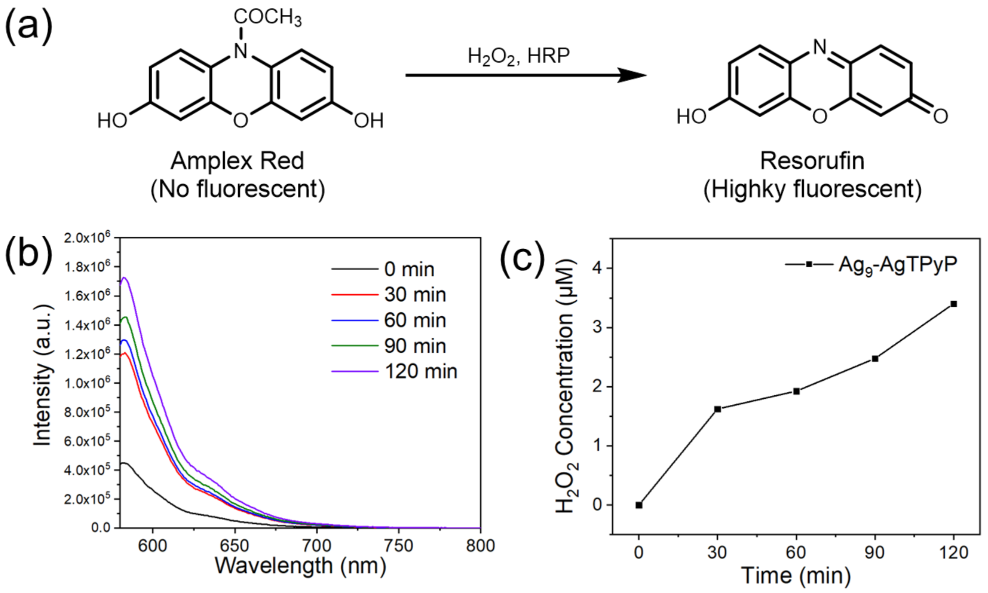

Figure S13. (a) Equations for the oxidation of N-acetyl-3,7-dihydroxyphenoxazine (Amplex red). (b) Time-dependent fluorescence spectra of Amplex Red for H_2O_2 detection. (c) H_2O_2 accumulation over time of Ag_9-AgTPyP.

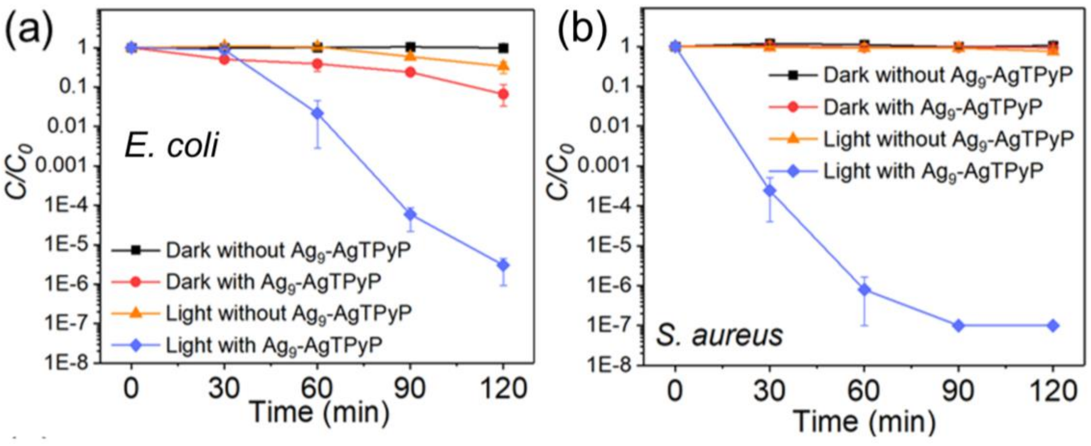

Figure S14. The antibacterial kinetics of the action of Ag_9-AgTPyP on (A) *E. coli* and (B) S. aureus.

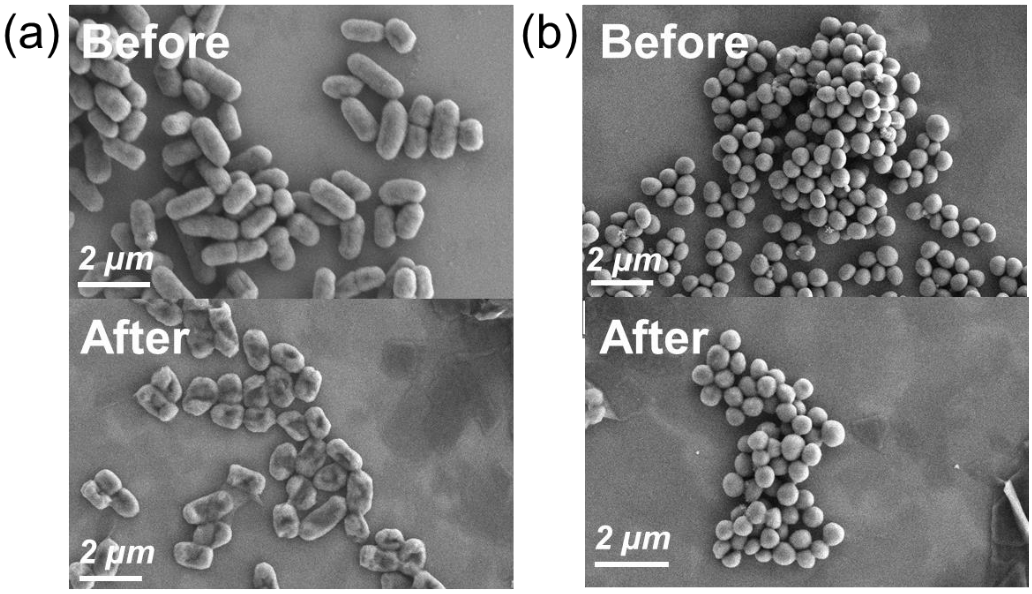

Figure S15. SEM images of (a) *E. coli*. and (b) *S.aureus* before and after photocatalytic disinfection with Ag_9-AgTPyP.

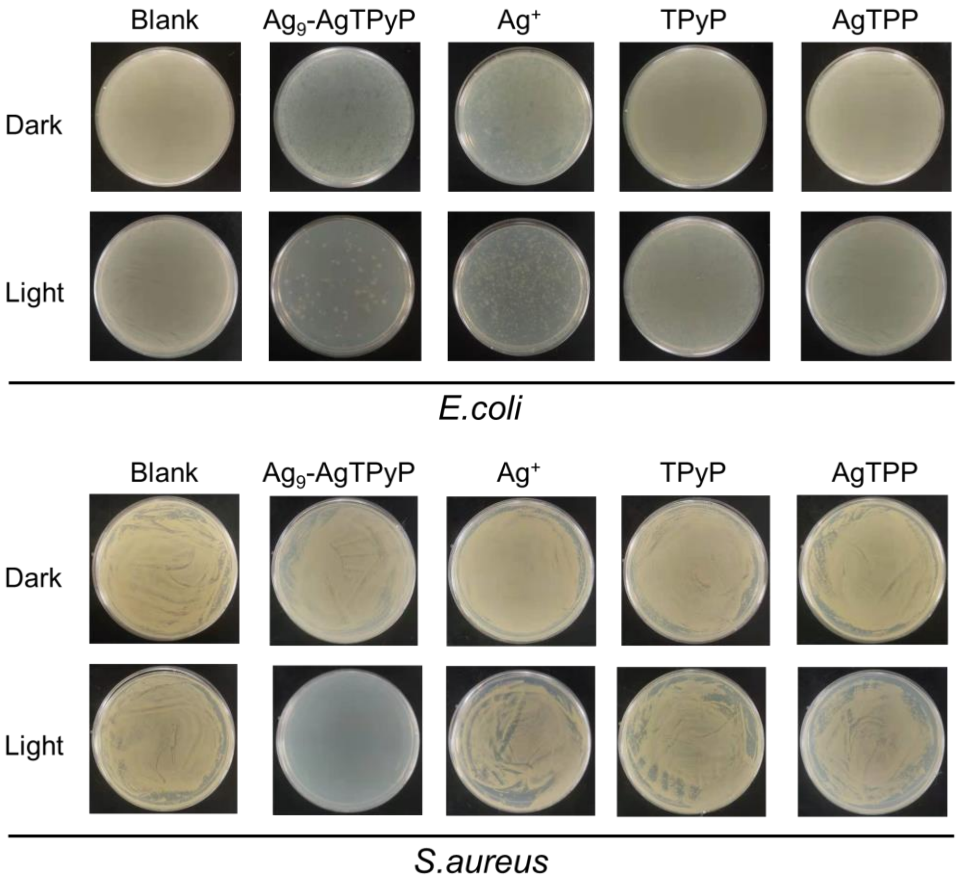

Figure S16. Photos of plate count agars spread with *E. coli* and *S.aureus* before and after photocatalytic disinfection using Ag_9-AgTPyP (0.5 mg), Ag^+, TPyP and AgTPP.

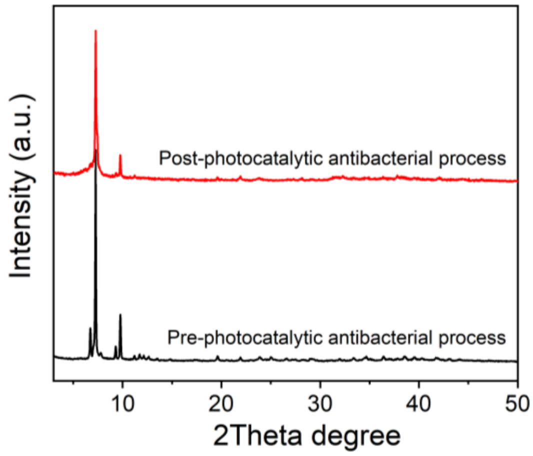

Figure S17. Post and pre- photocatalytic antibacterial process PXRD patterns of Ag_9-AgTPyP.

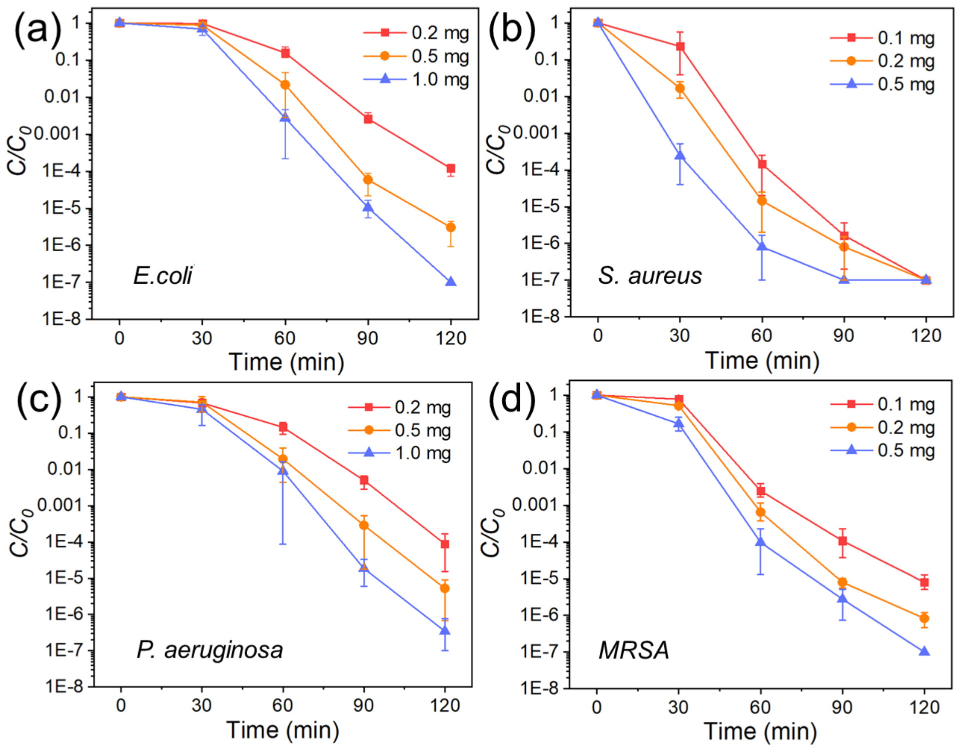

Figure S18. Optimization of modification mass of Ag_9-AgTPyP for antibacterial tests.

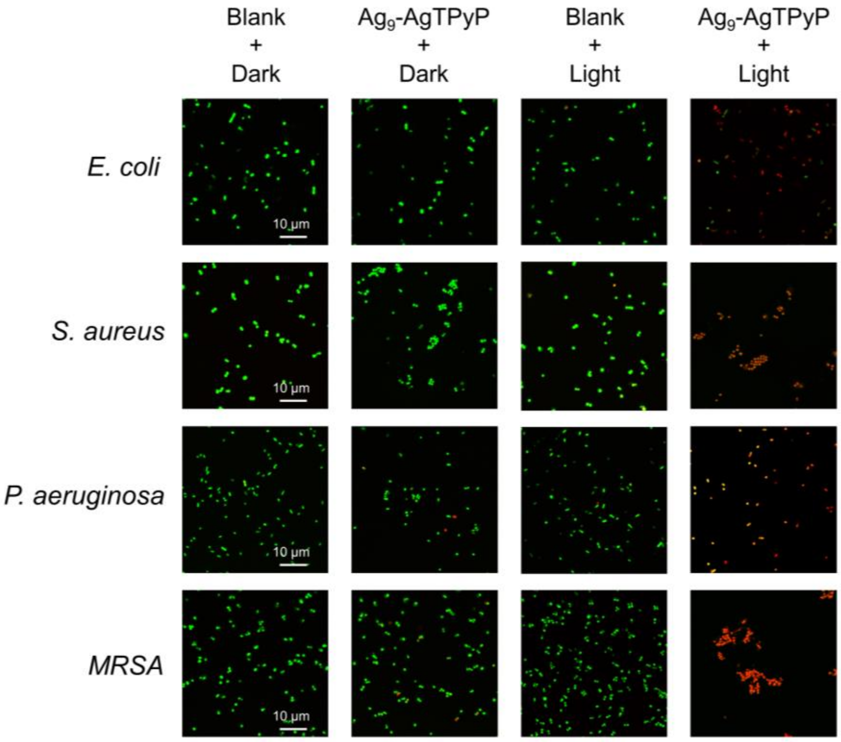

Figure S19. Fluorescence images of all bacterial (green) and dead bacterial (red) cells upon various treatment.

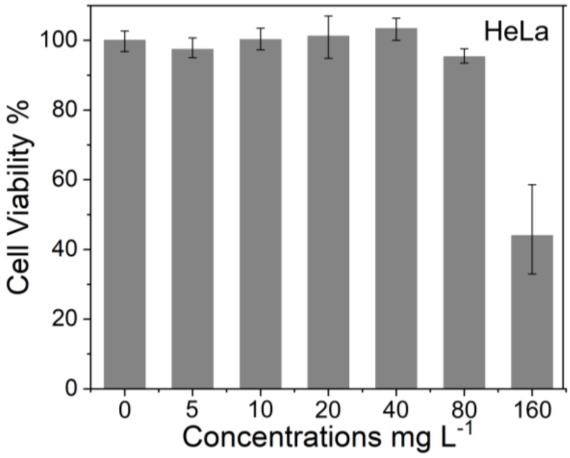

Figure S20. The cell cytotoxicity in vitro.

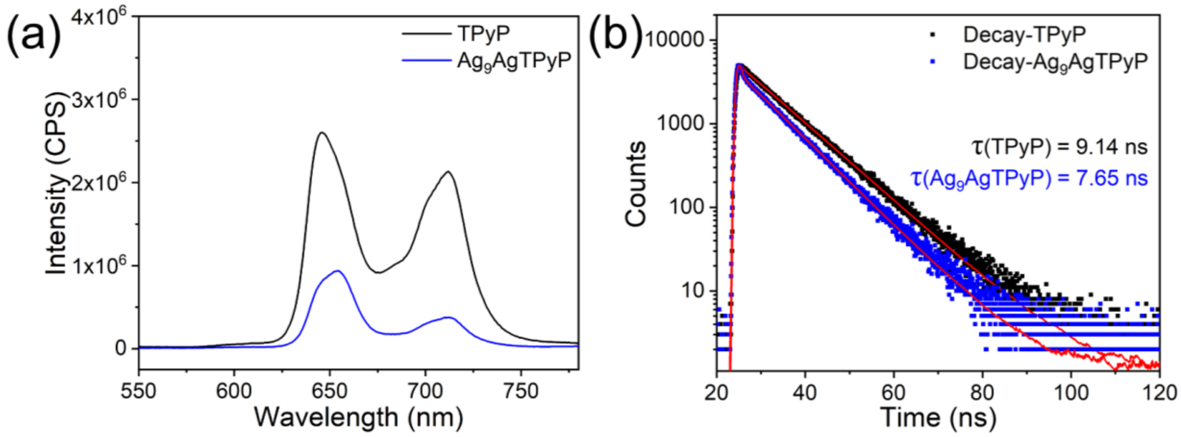

Figure S21. (a) Photoluminescence (PL) emission upon excitation at 405 nm and (b) time-resolved PL decay probed at 646 nm for TPyP and Ag_9-AgTPyP dispersed in EtOH.

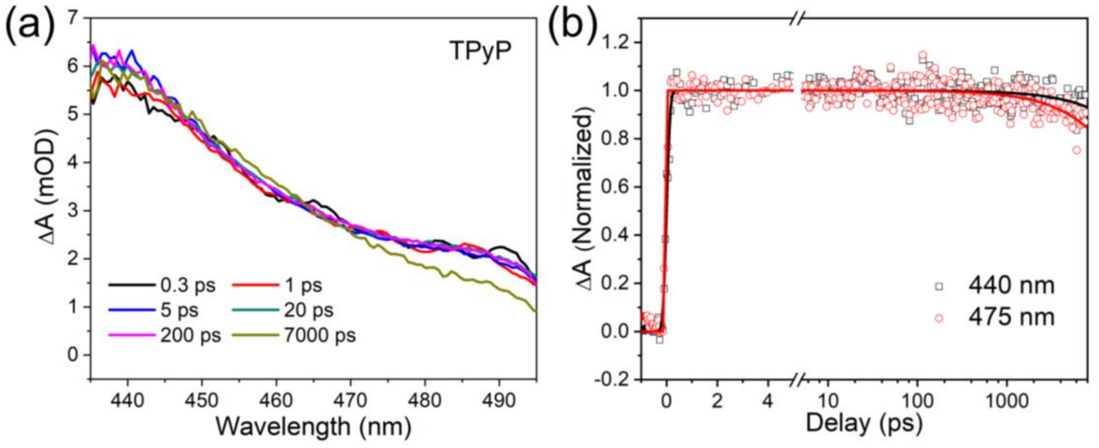

Figure S22. (a) TA spectra of TPyP registered at different probe delays (pump at 520 nm). (b) Representative TA kinetics of TPyP taken at the probing wavelength of 440 nm (black line) and 475 nm (red line).

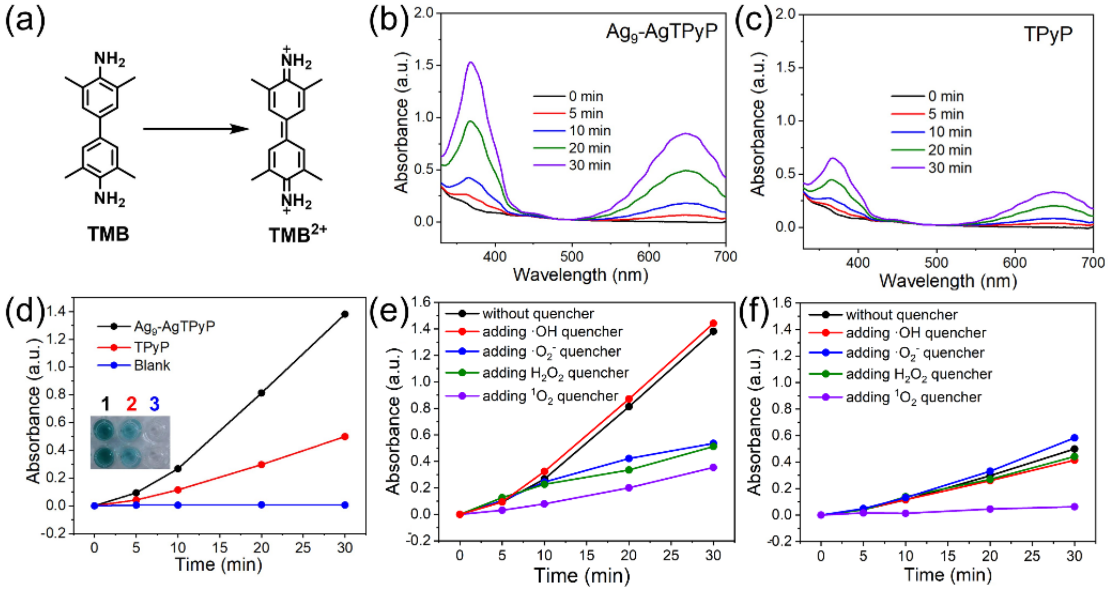

Figure S23. (a) The oxidation reaction equation of TMB. Time-dependent UV-vis absorption spectra recording TMB oxidation over (b) Ag_9-AgTPyP and (c) TPyP. (d) Comparison of oxidaselike activity of 1 of concentration 1 μM, the equivalent concentrations of the ligand molecules (1 μM). (Inset) Images of the corresponding solutions of (1) Ag_9-AgTPyP, (2) TPyP, (3) Blank. UV-vis absorbance for TMB oxidation product monitored at 370 nm along with reaction time over. (e) Ag_9-AgTPyP and (f) TPyP in the presence of different scavengers in air atmosphere under visible light irradiation.

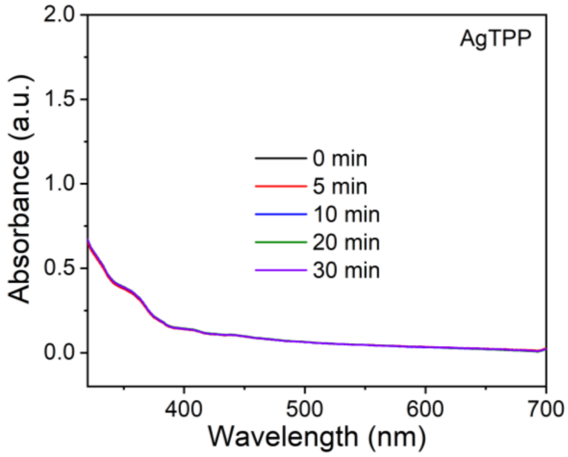

Figure S24. Time-dependent UV-vis absorption spectra recording TMB oxidation over AgTPP.

We have conducted the TMB oxidation experiment of AgTPP. As shown in above, AgTPP exhibited negligible activity within 30 min. The results clearly indicated that the central Ag ions in porphyrin rings could not enhance the ROS production efficiency, which is consistent with TA results.

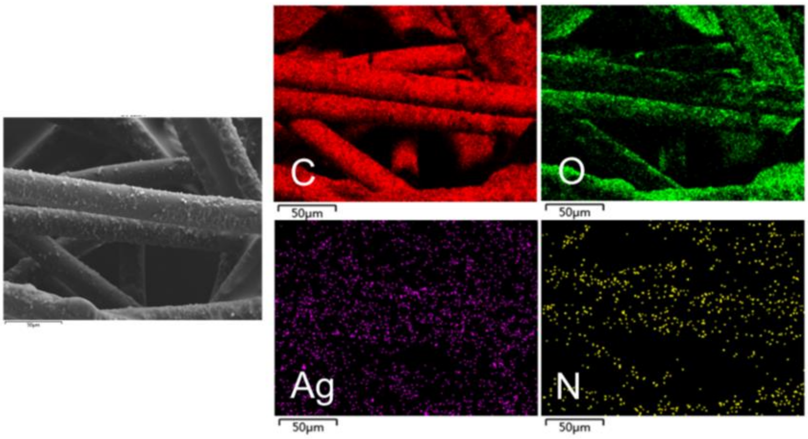

Figure S25. Elemental mapping analysis of Ag_9-AgTPyP.

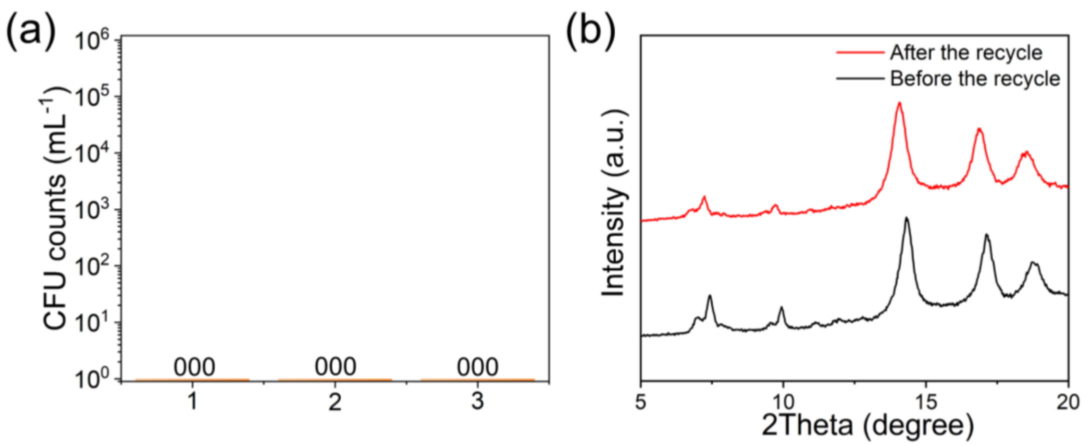

Figure S26. (a)Recycling test of Ag_9-AgTPyP film. (d) After and before the recycle patterns of Ag_9-AgTPyP film. The data marked by three zeros (000) on the bar indicate that no live bacteria were detected.

For traditional silver nanoparticles (AgNPs), they killed bacteria by releasing sliver ions (Ag^+), which further induced membrane damage, produced ROS or was untaken by cells with consequent disruption of Adenosine triphosphate (ATP) production and hindering of DNA replication activities. [1] The Ag^+ release efficiency of AgNPs is limited under air conditions. Moreover, the high surface energy of AgNPs often suffered from some aggregation issues, probably leading to inferior Ag^+ utilization efficiency. In contrast, Ag_9-AgTPyP used in our work, served as heterogeneous photocatalytic antibacterial material, which are functioning by producing more efficient ROS (including $^1O_2$, O_2^- and H_2O_2), which subsequently caused more lethal damage to bacteria, rather than releasing Ag^+.

Table S1. Crystal data and structure refinements for Ag_9-AgTPyP.

Compounds	Ag_9-AgTPyP
CCDC numbers	2054440
Empirical formula	$C_{82}H_{78}Ag_{10}F_9N_8O_6$
Formula weight	2521.22
Temperature/K	149(2)
Crystal system	monoclinic
Space group	*C2/c*
a/Å	24.5697(8)
b/Å	19.2501(5)
c/Å	22.6181(6)
α/°	90
β/°	98.112(3)
γ/°	90
Volume/Å^3	10590.6(5)
Z	4
ρ_{calc} g/cm^3	1.581
μ/mm^{-1}	14.999
F(000)	4900.0
Radiation	CuKα (λ = 1.54184)
2Θ range for data collection/°	6.77 to 124.976
Index ranges	$-28 \le h \le 24$, $-22 \le k \le 17$, $-25 \le l \le 26$
Reflections collected	21833
Independent reflections	8378 [R_{int} = 0.0641, R_{sigma} = 0.0792]
Goodness-of-fit on F^2	1.072
Final R indexes [I>=2σ (I)]	R_1 = 0.0839, wR_2 = 0.2462
Final R indexes [all data]	R_1 = 0.0960, wR_2 = 0.2568
Largest diff. peak/hole / e Å^{-3}	1.99/-1.57

[a] $R_1 = \Sigma||F_o| - |F_c||/\Sigma|F_o|$. [b] $wR_2 = |\Sigma w(|F_o|^2 - |F_c|^2)|/\Sigma|w(F_o^2)^2|^{1/2}$.

Table S2. The selected bond lengths (Å) for Ag_9-AgTPyP.

Ag1	Ag3#1	2.8787(10)	C34	C33	1.448(15)
Ag1	Ag3	2.8787(10)	C34	C35	1.358(14)
Ag1	Ag2	2.8963(9)	C33	C28	1.393(14)
Ag1	Ag2#1	2.8964(9)	N1	Ag1#5	2.389(8)
Ag1	C1	2.193(9)	N1	C27	1.264(15)
Ag1	C1#1	2.193(9)	N1	C23	1.333(17)
Ag1	N1#2	2.389(8)	N2	C41	1.343(16)
Ag1	N1#3	2.389(8)	N2	C40	1.283(17)
Ag6	N3	2.088(8)	C30	C29	1.478(13)
Ag6	N3#4	2.088(8)	C29	C28	1.398(14)
Ag6	N4#4	2.076(8)	C2	C3	1.518(9)
Ag6	N4	2.076(8)	C13	Ag4#1	2.134(15)
Ag3	Ag2#1	3.0904(12)	C13	C14	1.189(9)
Ag3	Ag2	2.9740(14)	C38	C42	1.351(17)
Ag3	Ag4#1	2.9812(12)	C38	C39	1.396(17)
Ag3	Ag5	2.9702(15)	C7	C8	1.19(2)
Ag3	C1	2.200(9)	O2	Ag3#1	2.411(13)
Ag3	C13	2.309(15)	O2	C19	1.416(10)
Ag3	O2#1	2.411(13)	C26	C27	1.383(17)
Ag2	Ag3#1	3.0902(12)	C24	C23	1.388(19)
Ag2	Ag4	2.9845(11)	O1	C19	1.203(9)
Ag2	Ag5	2.9519(16)	C42	C41	1.368(17)
Ag2	C1	2.235(10)	C40	C39	1.364(18)
Ag2	C7	2.256(15)	C19	C20	1.529(10)
Ag2	O1	2.291(12)	C14	C15	1.525(10)
Ag4	Ag3#1	2.9811(12)	C3	C4	1.523(9)
Ag4	Ag5#1	2.8830(18)	C3	C5	1.524(9)
Ag4	Ag5	2.8823(16)	C3	C6	1.533(9)
Ag4	N2	2.361(9)	F2	C20	1.385(10)
Ag4	C13#1	2.134(15)	F1	C20	1.387(10)
Ag4	C7	2.159(16)	F3	C20	1.393(10)
Ag5	Ag4#1	2.8830(18)	C9	C12	1.528(6)
Ag5	C13	2.175(18)	C9	C8	1.532(10)
Ag5	C7	2.17(2)	C9	C10	1.527(7)
Ag5	O3	2.241(8)	C9	C11	1.533(7)
N3	C36	1.353(12)	C15	C17	1.520(7)

N3	C33	1.378(13)	C15	C16	1.522(7)
N4	C32	1.351(13)	C15	C18	1.520(7)
N4	C29	1.351(13)	O3	C21	1.386(9)
C36	C37	1.421(13)	O3	C21#1	1.386(9)
C36	C35	1.430(15)	C21	O3#1	1.386(9)
C32	C31	1.443(13)	C21	C22	1.528(10)
C32	C37#4	1.438(14)	C22	C21#1	1.528(10)
C1	C2	1.202(8)	C22	F6	1.382(8)
C31	C30	1.317(16)	C22	F6#1	1.382(8)
C25	C28	1.487(13)	C22	F5	1.383(7)
C25	C26	1.308(16)	C22	F5#1	1.383(7)
C25	C24	1.323(17)	C22	F4	1.372(7)
C37	C32#4	1.438(14)	C22	F4#1	1.372(7)
C37	C38	1.480(14)	F4	F6#1	0.83(4)

#1 1-X,+Y,1/2-Z; #2 +X,-1+Y,+Z; #3 1-X,-1+Y,1/2-Z; #4 3/2-X,3/2-Y,1-Z; #5 +X,1+Y,+Z

Table S3. A summary of photoactive antibacterial material.

Sample	Key Component	Initial bacterial concentration (CFU mL^{-1})	Type of bacteria and $log_{10}(C_0/C)$ Efficiency	Size and concentration	References
TCP-C_{60}	Porphyrin, C_{60}	10^4	*E. coli* (4 in 60 min)<br>*S. aureus* (4 in 30 min)	0.09 cm^2 film	[2]
MMNPs	Porphyrin, HSA, Mn	10^6	*E. coli* (2 in 15 min)<br>*S. aureus* (1 in 15 min)	30 mg L^{-1} + 100 µM H_2O_2	[3]
CNC-Por	Zn-Porphyrin	10^8	*E. coli* (1.5 in 60 min)<br>*S. aureus* (6 in 60 min)	366 mg L^{-1}	[4]
CS/Ag/MoS_2-Ti	Cs, Ag, MoS_2, Ti	*E. coli* (10^6)<br>*S. aureus* (10^8)	*E. coli* (2.5 in 20 min)<br>*S. aureus* (2 in 20 min)	100 mg L^{-1}	[5]
ZIF-8	Zn-MOF	10^7	*E. coli* (6 in 120 min)	500 mg L^{-1}	[6]
FLV-MoS_2	MoS_2	10^6	*E. coli* (5 in 120 min)	1.6 mg L^{-1} film	[7]
Cu–MoS_2	MoS_2, Cu	10^6	*E. coli* (5 in 20 min)	1.6 mg L^{-1} film	[7]
F-g-C_3N_4-30-EP	C_3N_4	10^6	*E. coli* (6 in 30min)	10 mg L^{-1} film	[8]
**Ag_9-AgTPyP**	**Porphyrin, Ag**	**10^8**	***E. coli* (5 in 120min)**<br>***S. aureus* (7 in 90min)**<br>***P. aeruginosa* (5 in 120min)**<br>***MRSA* (7 in 120min)**	**50 mg L^{-1}**	**This work**

References

[1] L. Rizzelloa, P. P. Pompa, *Chem. Soc. Rev.* **2014**, *43*, 1501-1518.
[2] M. B. Ballatore, J. Durantini, N. S. Gsponer, M. B. Suarez, M. Gervaldo, L. Otero, M. B. Spesia, M. E. Milanesio, E. N. Durantini, *Environ. Sci. Technol.* **2015**, *49*, 7456-7463.
[3] Q. Deng, P. Sun, L. Zhang, Z. Liu, H. Wang, J. Ren, X. Qu, *Adv. Funct. Mater.* **2019**, *29*, 1903018.
[4] E. Feese, H. Sadeghifar, H. S. Gracz, D. S. Argyropoulos, R. A. Ghiladi, *Biomacromolecules* **2011**, *12*, 3528-3539.
[5] M. Zhu, X. Liu, L. Tan, Z. Cui, Y. Liang, Z. Li, K. W. Kwok Yeung, S. Wu, *J. Hazard. Mater.* **2020**, *383*, 121122.
[6] P. Li, J. Li, X. Feng, J. Li, Y. Hao, J. Zhang, H. Wang, A. Yin, J. Zhou, X. Ma, B. Wang, *Nat. Commun.* **2019**, *10*, 2177.
[7] C. Liu, D. Kong, P. C. Hsu, H. Yuan, H. W. Lee, Y. Liu, H. Wang, S. Wang, K. Yan, D. Lin, P. A. Maraccini, K. M. Parker, A. B. Boehm, Y. Cui, *Nature Nanotech.* **2016**, *11*, 1098-1104.
[8] Z. Teng, N. Yang, H. Lv, S. Wang, M. Hu, C. Wang, D. Wang, G. Wang, *Chem* **2019**, *5*, 664-680.
